# MET and PTEN gene copy numbers and Ki-67 protein expression associate with pathologic complete response in ERBB2-positive breast carcinoma patients treated with neoadjuvant trastuzumab-based therapy

**DOI:** 10.1186/s12885-016-2743-x

**Published:** 2016-08-30

**Authors:** Benjamin C. Calhoun, Bryce Portier, Zhen Wang, Eugen C. Minca, G. Thomas Budd, Christopher Lanigan, Raymond R. Tubbs, Larry E. Morrison

**Affiliations:** 1 Department of Pathology, Cleveland Clinic, 9500 Euclid Avenue, Cleveland, OH 44195 USA; 2 Department of Hematology and Oncology, Cleveland Clinic, Cleveland, OH USA; 3 Present Address: Ventana Medical Systems, Inc, 1910 E. Innovation Park Dr, Tucson, AZ 85755 USA

**Keywords:** Breast cancer, Neoadjuvant, Biomarkers, ERBB2, HER2, Pathologic complete response (pCR), In situ hybridization (ISH)

## Abstract

**Background:**

Pathologic complete response (pCR) after neoadjuvant chemotherapy for breast cancer is associated with improved prognosis in aggressive tumor subtypes, including ERBB2- positive tumors. Recent adoption of pCR as a surrogate endpoint for clinical trials in early stage breast cancer in the neoadjuvant setting highlights the need for biomarkers that, alone or in combination, help predict the likelihood of response to treatment.

**Methods:**

Biopsy specimens from 29 patients with invasive ductal carcinoma treated with trastuzumab-based therapy prior to definitive resection and pathologic staging were evaluated by dual color bright field in situ hybridization (dual ISH) using probes for *MET*, *TOP2A*, *PTEN*, and *PIK3CA* genes, each paired with centromeric probes to their respective chromosomes (chromosomes 7, 17, 10, and 3). Ki-67 expression was assessed by immunohistochemistry (IHC). Various parameters describing copy number alterations were evaluated for each gene and centromere probe to identify the optimal parameters for clinical relevance. Combinations of ISH parameters and IHC expression for Ki-67 were also evaluated.

**Results:**

Of the four genes and their respective chromosomes evaluated by ISH, two gene copy number parameters provided statistically significant associations with pCR: *MET* gain or loss relative to chromosome 7 (AUC = 0.791, sensitivity = 92 % and specificity = 67 % at optimal cutoff, *p* = 0.0032) and gain of *PTEN* (AUC = 0.674, sensitivity = 38 % and specificity = 100 % at optimal cutoff, *p* = 0.039). Ki-67 expression was also found to associate significantly with pCR (AUC = 0.726, sensitivity = 100 % and specificity = 42 % at optimal cutoff, *p* = 0.0098). Combining gain or loss of *MET* relative to chromosome 7 with Ki-67 expression further improved the association with pCR (AUC = 0.847, sensitivity = 92 % and specificity = 83 % at optimal cutoffs, *p* = 0.0006).

**Conclusions:**

An immunogenotypic signature of low complexity comprising *MET* relative copy number and Ki-67 expression generated by dual ISH and IHC may help predict pCR in ERBB2-positive breast cancer treated with neoadjuvant chemotherapy and trastuzumab. These findings require validation in additional patient cohorts.

**Electronic supplementary material:**

The online version of this article (doi:10.1186/s12885-016-2743-x) contains supplementary material, which is available to authorized users.

## Background

Pathologic complete response (pCR) after neoadjuvant chemotherapy for breast cancer is associated with improved prognosis [1]. The prognostic value of a pCR may be greatest in aggressive tumor subtypes, including ERBB2-positive tumors [1]. The Food and Drug Administration (FDA) has issued guidance on the use of pCR as a surrogate endpoint for clinical trials in early stage breast cancer in the neoadjuvant setting [2]. Pertuzumab, an inhibitor of heterodimerization of ERBB2 (erb-b2 receptor tyrosine kinase 2, commonly known as HER-2 and HER-2/*neu*) with other ERBB receptor family members, is the first agent granted accelerated approval for the neoadjuvant treatment of high-risk early stage breast cancer based on pCR data [3]. Given the importance of pCR in prognosis and clinical trial design, there is a need to identify biomarkers that, alone or in combination, help predict the likelihood of response to treatment. A variety of genes, including *PIK3CA*, *PTEN*, *TOP2A* and *MET* are candidate markers for prognosis and response to treatment in ERBB2-positive breast cancer.

Genetic alterations in the phosphatidylinositol 3-kinase (PI3K)/V-AKT murine thymoma viral oncogene homolog (AKT)/mechanistic target of rapamycin (MTOR) pathway are common events in breast cancer [4, 5]. Preclinical data in cell lines indicate that mutations in the p110 alpha-catalytic subunit of PI3K (PIK3CA) lead to resistance to trastuzumab and lapatinib [6–9]. Several clinical studies have examined the association between somatic mutations in *PIK3CA* and benefit from ERBB2-targeted therapy [10–14]. In the FinHer [10] and NSABP B-31 adjuvant trials [11], there was no significant loss of trastuzumab efficacy observed in patients with *PIK3CA* mutations. In the NeoALLTO neoadjuvant trial which incorporated lapatinib as well as trastuzumab, patients with *PIK3CA* mutations were less likely to have a pCR, but there were no significant differences in progression-free or overall survival [12]. Other neoadjuvant trials have shown similar results [13, 14]. Comparatively little is known about *PIK3CA* gene copy number alterations and clinical outcomes in breast cancer, irrespective of ERBB2 status [15]. Amplification of mutant *PIK3CA* alleles appears to contribute to resistance to PI3K inhibitors in preclinical breast cancer models [16].

Phosphatase and tensin homolog (PTEN) is the 3’ lipid phosphatase for phosphatidylinositol-3,4,5-triphospate (PIP3), thereby negatively regulating downstream signaling by PIP3 after phosphorylation by PI3K [17, 18]. Patients with ERBB2-positive, PTEN-deficient tumors may develop resistance to ERBB2-targeted therapy [6, 9, 19–22]. Many small, retrospective studies have shown that PTEN deficiency or absence may be associated with reduced clinical benefit from trastuzumab [19, 20, 23, 24]. However, the recent data from a large prospective study of early stage ERBB2-positive breast cancer indicate that patients with and without PTEN deficiency by immunohistochemistry derived benefit from treatment with trastuzumab [25]. PTEN status in most studies was determined by immunohistochemistry or gene sequencing and relatively little is known about the significance of *PTEN* copy number alterations in response to ERBB2-targeted therapy.

The topoisomerase II alpha (TOP2A) and *ERBB2* genes are located close to each other on the long arm of chromosome 17 and may be co-amplified in breast cancer [26–29]. Approximately 35 % of ERBB2-amplified tumors show *TOP2A* gene amplification [30, 31] and deletions are much less common [30, 32]. Alterations in *TOP2A* copy number have mainly been associated with response to anthracycline-based chemotherapy [30, 33]. The role of *TOP2A* amplification or deletion in response to ERBB2-targeting has not been thoroughly investigated.

Overexpression of MET proto-oncogene, receptor tyrosine kinase (MET) occurs in 20 % – 30 % of invasive breast cancers [34] and is associated with a poor prognosis in lymph node-positive and lymph node-negative disease and across all molecular subtypes [35–40]. In the metastatic setting, increased *MET* copy numbers correlate with trastuzumab therapy failure in ERBB2-positive breast cancer [41] and clinical trials with anti-MET therapy in advanced breast cancer are ongoing [42]. The significance of *MET* amplification or deletion in the response to adjuvant or neoadjuvant therapy for ERBB2-positive breast cancer is not well established.

In this exploratory study using in situ hybridization (ISH) and immunohistochemistry (IHC), we assessed alterations in gene copy number for *PIK3CA*, *PTEN*, *TOP2A* and *MET*, and their respective chromosomes, and expression of Ki-67 in a series of patients with ERBB2-positive tumors who were treated with chemotherapy and trastuzumab in the neoadjuvant setting. Various parameters representing gene and chromosome copy numbers were evaluated for association with pCR in an effort to identify parameters most effective for improving prediction in the neoadjuvant treatment of ERBB2-positive breast cancer.

## Methods

This study was approved by the Cleveland Clinic Institutional Review Board. All patients who received trastuzumab at the Cleveland Clinic from January 2008 to December 2010 were reviewed for study inclusion (234 patients). Of the 234 cases, 29 satisfied inclusion criteria which included a diagnosis of primary invasive breast cancer, neoadjuvant trastuzumab therapy, and a pre-treatment biopsy performed at the Cleveland Clinic. Pathology data was obtained from the Anatomic Pathology information system CoPath Plus (Cerner Corporation, Kansas City, MO). Clinical data was obtained from the electronic medical record Epic (Epic Systems Corporation, Verona, WI). The age, tumor size, pre-treatment clinical stage, hormone receptor status by IHC, *ERBB2* status by ISH, post-treatment pathologic stage, and presence or absence of a pCR were recorded for all patients (Table 1).

**Table 1:** Clinical and pathologic characteristics of patients with ERBB2-positive breast cancer treated with neoadjuvant chemotherapy and trastuzumab

Case ID	Size, largest (mm)	Clinical TNM	Clinical stage	ER IHC	PR IHC	HER2 copy number (Average)	HER2/CEP17 Ratio	Pathologic Stage	pCR
1	49	cT2N2M0	IIIA	pos	pos	9.1	4.1	ypT0N0	yes
2	47	cT2N0M0	IIA	neg	neg	7.4^c^	3.7^c^	ypTisN0	yes
3	37	cT2N0M0	IIA	neg	neg	18.5	8.4	ypTisN0	yes
4	12	cT1N1M0	IIA	pos	neg	19.2	9.6	ypT0N0	yes
5	60	cT3N1M0	IIIA	pos	neg	8.1	3.7	ypTisN0	yes
6	63	cT3N1M0	IIIA	neg	neg	20.0	11.1	ypT0N0	yes
7	20	cT4N1M0	IIIB	neg	neg	17.2	6.6	ypT0N0	yes
8	36	cT2N1M0	IIB	neg^a^	neg^a^	17.4	6.7	ypT0N0	yes
9	18	cT1N2M0	IIIA	pos	pos	11.3	4.5	ypT0N0	yes
10	28	cT4N1M0	IIIB	pos	pos	15.8	7.5	ypT0N0	yes
11	47	cT2N0M0	IIA	neg^a^	neg^a^	4.3	2.9	ypT0Nx	yes
12	36	cT2N0M0	IIA	neg	neg	13.2	5.5	ypT0N0	yes
13	37	cT4N0M0	IIIB	pos	pos	20.0	16.7	ypT0N0	yes
14	42	cT2N1M0	IIB	pos	neg	3.3	2.4	ypT0N0	yes
15	60	cT3N0M0	IIB	pos	pos	14.7	8.2	ypT0N0	yes
16	20	cT1N1M0	IIA	pos	pos	4.9	3.1	ypTisN1	no
17	60	cT4N1M0	IIIB	pos	pos	5.0	3.1	ypT3N0	no
18	64	cT3N0M0	IIB	pos	pos	16.1	14.6	ypT1miN0	no
19	51	cT3N1M0	IIIA	pos	pos	5.2	3.2	ypT1N0	no
20	40	cT2N1M0	IIIA	pos	pos	4.4	2.3	ypT2N1	no
21	21	cT2N1M0	IIIA	pos	pos	16.6	8.3	ypT1N0	no
22	35	cT4N1M0	IIIB	pos	neg^a^	5.3	2.9	ypT3N0	no
23	86	cT3N1M0	IIIA	pos	pos	5.0	3.2	ypT1N1	no
24	27	cT2N1M0	IIIA	neg	neg	15.9	7.2	ypT1N2	no
25	43	cT2N1M0	IIIA	pos	pos	14.0	5.6	ypT1N0	no
26	60	cT4N2M0	IIIB	pos	pos	16.7	8.4	ypT3N1	no
27	29	cT2N2M0	IIIA	pos	pos	16.2	10.1	ypT1N2	no
28	73	cT3N0M0	IIB	pos	pos	9.1	3.5	ypT2N1	no
29	41	cT2N0M0	IIA	neg	neg	3.6^b^	1.8^b^	ypT1N1	no

Abbreviations: *ER* estrogen receptor, *IHC* immunohistochemistry, *PR* progesterone receptor, *pCR* pathologic complete response

^a^Cases reported as negative, < 5 %, prior to the 2010 ASCO/CAP Guidelines

^b^ERBB2 immunohistochemistry was 3+

^c^ *ERBB2* genetic heterogeneity present; average ERBB2 copy number and ERBB2/CEP17 ratio reported for amplified clone

### Patients and clinical assessment

Formalin-fixed paraffin-embedded (FFPE) needle biopsy specimens from early stage breast cancer patients treated in the neoadjuvant setting were obtained from the archives of the Cleveland Clinic (Cleveland, OH). Chart review and study analyses were approved by the Cleveland Clinic institutional review board. Eligibility criteria for further evaluation included histologic confirmation of clinical stage IIA to IIIB, ERBB2 amplification by in fluorescent in situ hybridization (FISH), and neoadjuvant treatment that included trastuzumab. Post-treatment pathologic staging was obtained from pathology reports and confirmed by histologic evaluation. For this study, classification as pCR required the absence of any detectable invasive carcinoma in the breast specimen and the axillary lymph nodes (i.e., ypT0N0 and ypTisN0).

With respect to treatment, of the 29 patients in the cohort 11 were treated with anthracycline-based chemotherapy including cyclophosphamide, a taxane, and trastuzumab (i.e. ACTH) and 18 received a taxane and trastuzumab with or without carboplatin (TCH). Of the 15 patients with a pCR, 5 were treated with ACTH and 10 were given TCH. Of the 14 patients who did not have a pCR, 6 received ACTH and 8 were treated with TCH. A total of 12 patients presented with clinical stage IIA or IIB disease and 8 of these patients had a pCR. Of the 8 patients with a pCR, 3 were treated with ACTH and 5 were given TCH. Of the 4 stage II patients who did not have a pCR, 1 was treated with ACTH and 3 were given ACTH. In this small exploratory study there may be some imbalance in the distribution of stage II patients in the two treatment groups. However, we believe the distribution of patients treated with ACTH versus TCH is relatively well-balanced among those who did or did not have a pCR.

### Immunohistochemistry

Ki-67 automated IHC was performed on 3–6 μm thick sections of FFPE specimen blocks using primary antibody 30-9 with iVIEW detection on the VENTANA BenchMark XT automated stainer instrument (all reagents and instrument from Ventana Medical Systems, Tucson, AZ) using the company recommended protocols.

### Silver and chromogenic in situ hybridization (SISH and CISH)

Automated in situ hybridization (ISH) was performed on 3–6 μm thick sections of FFPE specimen blocks using the Ventana Medical Systems dual ISH procedure (dual color dual hapten DNA in situ hybridization) on the BenchMark XT automated stainer. Probes were hybridized in the following pairs, each comprising a gene locus probe, referred to by the name of a gene contained within the targeted region (e.g. *MET*), and a centromere (CEN) probe for the chromosome on which the gene locus lies (e.g. CEN7): *MET* + CEN7, *TOP2A* + CEN17, *PTEN* + CEN10, and *PIK3CA* + CEN3. All gene probes were detected using peroxidase-catalyzed silver staining (SISH) and centromeric probes were detected using chromosome staining with alkaline phosphatase-catalyzed fast red staining (CISH). ISH probes and associated detection reagents are commercially available from Ventana Medical Systems.

### Fluorescent in situ hybridization (FISH)

*ERBB2* status determination was performed using an FDA-approved interphase FISH assay (PathVysion®, Abbott Molecular, Des Plaines, IL). Consistent with the timeframe in which the patients were treated, *ERBB2* scoring methods were applied to FISH samples in accordance with the 2007 ASCOCAP guidelines [43]. Briefly, ASCOCAP dual-probe scoring was applied as follows: non-amplified (*ERBB2*CEP17 < 1.8), equivocal (*ERBB2*CEP17 1.8–2.2), or amplified (*ERBB2*CEP17 > 2.2). For the cases in which it was performed, ERBB2 IHC (4B5, Ventana Medical Systems Inc, Tucson, AZ) was scored according to the 2007 ASCOCAP guidelines [43] as 0, 1, 2, or 3.

### Specimen evaluation

Hybridized and coverslipped specimens were viewed with brightfield microscopy to enumerate the SISH (metallic silver - black) gene locus signals and CISH (red) centromere signals on a cell-by-cell basis. Only invasive carcinoma tumor cells were selected for cell-by-cell signal enumeration using a 40X objective in combination with 10X eyepieces. In general, 50 invasive tumor cells were enumerated per specimen except in several specimens for which fewer than 50 cells with good hybridization signals could be found (a minimum of 20 cells were required for inclusion in the analysis). Gene locus and centromere copy number statuses were assessed using a number of different parameters, including:

1. the average number of gene or centromere copies per cell, designated as gene/cell (e.g. *MET*/cell) or centromere/cell (e.g. CEN7/cell),
2. the percentage of cells with greater than 2 gene or centromere signals, designated as gene gain (e.g. *MET* gain) or centromere gain (e.g. CEN7 gain),
3. the percentage of cells with less than 2 gene or centromere signals, designated as gene loss (e.g. *MET* loss) or centromere loss (CEN7 loss),
4. the percentage of cells with either greater than 2 or less than 2 gene locus or centromere signals, designated as gene gain or loss (e.g. *MET* gain or loss), or centromere gain or loss (e.g. CEN7 gain or loss),
5. the average number of gene copies per corresponding centromere copies, designated as gene locus/centromere (e.g. *MET*/CEN7),
6. the percentage of cells with more copies of the gene than the corresponding centromere, designated as gene/centromere gain (e.g. *MET*/CEN7 gain),
7. the percentage of cells with fewer copies of the gene than the corresponding centromere, designated as gene/centromere loss (e.g. *MET*/CEN7 loss), and
8. the percentage of cells with either more copies of the gene than the corresponding centromere or fewer copies of the gene than the corresponding centromere, designated as gene/centromere gain or loss (e.g. *MET*/CEN7 gain or loss).

The average number of genes or centromeres per cell was calculated by summing the number of gene or centromere signals over all the cells enumerated within a specimen, and dividing by the number of cells enumerated. The average number of genes per centromere was calculated by summing the number of gene signals over all the cells enumerated and dividing by the sum of the centromere signals in all of the cells enumerated.

For Ki-67 IHC staining interpretation was performed only for invasive tumor cells and was evaluated by selecting the area of invasive carcinoma with the highest proliferation rate and then determining the percentage of approximately 50 invasive tumor cell nuclei that were positive for Ki-67 expression. The cutoff for Ki-67 positively staining cells was determined empirically to provide the best combined sensitivity and specificity for pCR in this neoadjuvant setting. An additional data file (.xls) lists the values for each ISH and IHC parameter described above for each patient in the study [Additional file 1].

For each parameter a range of cutoffs was evaluated such that specimens with the parameter value greater than or equal to a cutoff were considered ‘high’ for that parameter and specimens with the parameter value less than the cutoff were considered ‘low’ for that parameter. Sensitivities and specificities for detecting patients with pCR, based on either the high parameter being positive for pCR or based on the low parameter being positive for pCR, were calculated for each parameter and each cutoff. Receiver Operating Characteristics (ROC) curves were generated as sensitivity versus 1 - specificity over all cutoffs tested, and Area Under the Curve (AUC) was calculated for each curve as one measure of a parameter’s ability to distinguish patients with pCR from patients without pCR, with AUC = 1 being ideal and progressively lower values being less favorable. AUC values near 0.5 indicate no ability to distinguish between patients. In addition to ROC analysis, 2X2 contingency tables were evaluated at each cutoff and probabilities from Fischer’s Exact test were used to gauge the statistical significance of the association between the binarized parameter (high versus low values) and pCR. The optimal cutoff for a parameter was the cutoff value providing the best combined sensitivity and specificity, which typically provided the lowest *p*-value.

In addition to the various single parameters, a ROC curve for the combinations of *MET*/CEN7 gain or loss with Ki-67 expression was generated by using the cutoff providing optimal sensitivity and specificity for *MET*/CEN7 gain or loss while varying the cutoff for Ki-67 over a wide range, with the combined parameters considered positive if both parameters were equal to or greater than the respective cutoff values, as described by Shultz, 1995 [44].

## Results

A total of 29 patients meeting inclusion criteria with tissue available for IHC and ISH studies were identified (Table 1). Of these patients, 27 had Ki-67 expression data, 24 had *MET* and CEN7 ISH counts, 25 had *PTEN* and CEN10 ISH counts, and 24 had both Ki-67 expression data and *MET* and CEN7 ISH counts. The mean and median age at presentation was 53 and 52 years, respectively. The mean and median pre-treatment tumor size was 49 and 41 mm, respectively (range = 12−86). Patients who presented with Stage IV disease and who underwent breast surgery were excluded. Overall, 15 of 29 (52 %) patients had a pCR, defined as ypT0/ypTis N0. Among the 14 patients who did not have a pCR, the residual invasive tumor measured less than 1 mm in 1 patient and 1 patient had residual ductal carcinoma in situ (DCIS) and a positive lymph node (ypTisN1).

**Fig. 1:**
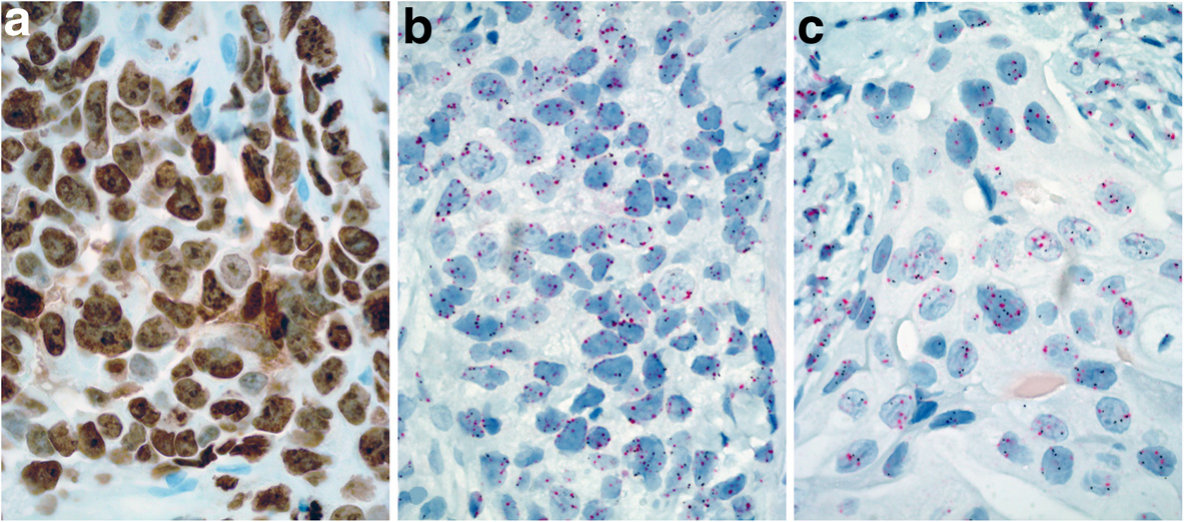
Representative images of immunohistochemistry and in situ hybridization studies from three tumors. **a**: Immunohistochemistry for Ki-67 showing positive staining in greater than 90 % of nuclei in a specimen from Case #2. **b**: ISH for *MET* + CEN7 showing reduced *MET* copy number [silver (black) signals] relative to chromosome 7 [red signals] in a specimen Case #2. **c**: ISH for *PTEN* + CEN10 showing gains in *PTEN* copy number [silver (black) signals] and chromosome 10 copy number [red signals] in a specimen from Case #3. (Original magnification x 600)

Gene copy numbers for *TOP2A*, *MET*, *PTEN*, and *PIK3CA*, and their corresponding chromosome copy numbers were measured by ISH, and Ki-67 protein expression was measured by IHC to identify associations with pCR individually and in combination. Figure 1, parts A, B, and C, show representative Ki-67 IHC, *MET* (black) + CEN7 (red) ISH, and *PTEN* (black) + CEN10 (red) ISH staining, respectively, on selected FFPE sections of biopsy specimens from the neoadjuvant breast cancer cohort. Figure 1a shows Case #2 (see Table 1 for characteristics of case #2) stained by IHC for Ki-67 and was determined to express Ki-67 in greater than 90 % of the cells. Figure 1b shows Case #2 stained for *MET* (black) and CEN7 (red) by ISH and shows cells with a lesser number of *MET* signals than CEN7 signals (48 % of cells showed relative *MET* loss). The tumor had other areas with a greater number of *MET* signals than CEN7 signals (34 % of cells showed relative *MET* gain) as well. Figure 1c shows Case #3 stained for PTEN (black) and CEN10 (red) with increased copy numbers for both loci (86 % of cells are near-tetrasomy).

**Fig. 2:**
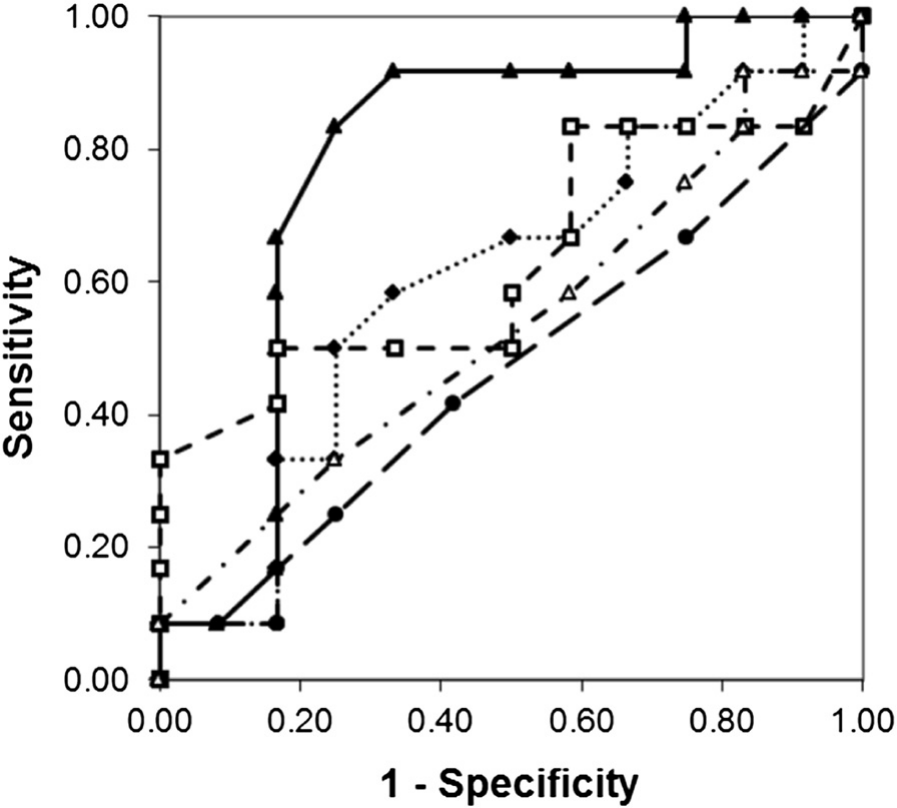
ROC curves for different parameters measuring *MET* gene copy number relative to chromosome 7 copy number. Parameters plotted include: *MET*/CEN7 gain or loss (solid line with solid triangles), *MET*/CEN7 gain (dotted line with solid diamonds), *MET*/CEN7 loss (short dashed line with open squares), *MET*/CEN7 (high ratios associated with pCR; long dashed line with solid circles), and *MET*/CEN7 (low ratios associated with pCR; alternating dashed and dotted line with open triangles)

ISH signals were interpreted using several different parameters to express gene and chromosome copy numbers in order to determine which parameters best associated with pCR. Figure 2 shows plots of 5 ROC curves, each representing a different parameter describing *MET* gene copy number relative to chromosome 7 copy number (see Methods section for a definition of each parameter). AUC values for each curve are listed in Table 2, along with the optimal cutoff values, whether the high parameter values (equal to or above the cutoff) or low parameter values (below the cutoff) were associated with pCR to generate the ROC curve and contingency analysis, the sensitivity and specificity obtained for those optimal cutoff values, and *p*-values using Fischer’s Exact test for the 2x2 contingency tables generated at those cutoffs. These ROC curves show a large difference between the association of the various *MET*/CEN7 parameters and pCR. The two ROC curves representing average *MET*/CEN7 counts, one associating ratios greater than the cutoff with pCR and the other associating ratios lower than the cutoff with pCR, show little if any association (AUC values near 0.5). The parameters representing the percentages of cells with more copies of *MET* than CEN7 (*MET*/CEN7 gain) and the percentages of cells with less copies of *MET* than CEN7 (*MET*/CEN7 loss) provided larger AUC values (0.613 and 0.651, respectively), while the parameter based on the sum of the percentage of cells with more copies and less copies of *MET* than CEN7 (*MET*/CEN7 gain or loss) provided the largest AUC value (0.791).

**Table 2:** Contingency table and ROC analysis results for parameter associations with pCR

Parameter	N, pCR	N, non-pCR	pCR correlated state^a^	ROC AUC	Optim c/o	Sens	Spec	*p*
*MET*/CEN7 gain or loss	12	12	high	0.791	50 % cells	0.92	0.67	0.0094
*MET*/CEN7 gain	12	12	high	0.613	32 % cells	0.58	0.67	0.41
*MET*/CEN7 loss	12	12	high	0.651	24 % cells	0.50	0.83	0.19
*MET*/CEN7	12	12	high	0.479	1.10	0.42	0.58	1.0
*MET*/CEN7	12	12	low	0.547	1.00	0.33	0.75	1.0
Ki-67	15	12	high	726	8 % cells	1.00	0.42	0.0098
*MET*/CEN7 gain or loss AND Ki-67	12	12	high/high	847^b^	50 % cells/8 % cells	0.92	0.83	0.0006
*PTEN* gain	13	12	high	674	58 % cells	0.38	1.00	0.039

Abbreviations: *N* number of specimens, *pCR* pathologic complete response, *AUC* area under curve, Optim *c/o* optimal cutoff (cutoff producing best combined sensitivity and specificity), *Sens* sensitivity, *Spec* specificity, *p* probability calculated using Fischer’s Exact test on contingency tables generated using optimal cutoff(s) as executed using JMP Statistical Software (SAS, Cary, NC)

^a^The parameter state that is associated with pCR in ROC curve and contingency table calculations, for which high state comprises specimens with parameter values equal to or greater than the optimum cutoff and low state comprises specimens with parameter values less than the optimum cutoff

^b^The AUC for the combined parameters equals the area under the ROC curve of *MET*/CEN7 gain or loss plus the additional area under the ROC curve of *MET*/CEN7 gain or loss, holding cutoff constant at 50 %, combined with Ki-67, varied across all possible cutoffs

**Fig. 3:**
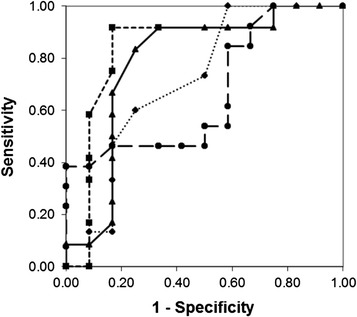
ROC curves for *MET*, Ki-67, PTEN, and *MET* combined with Ki-67 parameters. Parameters plotted include: *MET*/CEN7 gain or loss (solid line with solid triangles; repeated from Fig. 2), Ki-67 expression (dotted line with solid diamonds), *PTEN* gain (long dashed line with solid circles), and *MET*/CEN7 gain or loss held at a constant cutoff of 50 % of cells while varying the cutoff for Ki-67 expression (short dashed line with solid squares)

Other parameters showing associations with pCR included Ki-67 expression (AUC = 0.726) and the percentage of cells with greater than the normal 2 *PTEN* copies, *PTEN* gain (AUC = 0.674), the ROC curves of which are plotted in Fig. 3, and whose AUC values, optimal cutoffs, related sensitivities and specificities, and *p*-values are included in Table 2. The parameter *MET*/CEN7 gain or loss was further analyzed in combination with Ki-67 expression, the 2 parameters providing the largest AUC values alone. This was done by generating an ROC curve [44] in which the cutoff for *MET*/CEN7 gain or loss was held at its optimal value of 50 % cells while varying the cutoffs for Ki-67 expression (plotted in Fig. 3), requiring both parameters in the combination to be equal to or greater than their respective cutoffs for a positive designation. The additional AUC provided by the combination with Ki-67 was 0.056 over that of *MET*/CEN7 gain or loss alone. The optimal cutoff for Ki-67 expression in combination with *MET*/CEN7 gain or loss was 8 % cells, maintaining the sensitivity at 92 % while increasing the specificity from 67 to 83 %, and improving the *p*-value to 0.0006 (see Table 2). *PTEN* copy number was also evaluated in combination with Ki-67 expression but improvement in *p*-value and combined sensitivity and specificity over the individual parameters was less (data not shown).

Clinical and pathologic characteristics listed in Table 1 for each patient were compared to the ISH and IHC parameters in Table 2 that showed statistically significant associations with pCR, as well as compared to pCR, using contingency tables (stage, ER IHC, PR IHC) and t-tests (age, tumor size, *ERBB2*/cell, and *ERBB2*/CEN17). Results are listed in Table 3 and show few statistically significant associations. *MET*/CEN7 gain or loss and MET/CEN7 gain or loss combined with Ki-67 expression were significantly associated with age (trend with tumor size), Ki-67 expression was strongly associated with both *ERBB2*/cell and *ERBB2*/CEN17, and pCR was significantly associated with only PR expression (trends with ER expression, *ERBB2*/cell, and age).

**Table 3:** Associations between clinical and pathologic characteristics (Table 1) and ISH parameters and pCR

Parameter^a^		Mean age ± SD	Mean size, largest (mm) ± SD	Stage		ER IHC		PR IHC		Mean *ERBB2*/cell	Mean *ERBB2*/CEN17
				II	III	pos	neg	pos	neg		
*MET*/CEN7 gain or loss	high	59.3 ± 13.4	39.4 ± 16.6	8	7	9	6	5	10	10.6 ± 5.8	4.9 ± 2.3
	low	47.0 ± 13.0	51.2 ± 18.6	3	6	7	2	7	2	11.9 ± 5.6	6.9 ± 4.0
	p	0.041	0.14	0.42		0.66		0.09		0.59	0.19
Ki-67	high	54.8 ± 14.8	41.6 ± 15.4	9	13	14	8	11	11	13.1 ± 5.5	7.0 ± 3.8
	low	46.0 ± 10.7	57.2 ± 21.6	2	3	4	1	3	2	5.6 ± 2.0	2.9 ± 0.7
	p	0.16	0.19	1.00		0.64		1.00		<0.0001	<0.0001
*MET*/CEN7 gain or loss AND Ki-67	high	61.5 ± 12.9	37.2 ± 14.9	7	6	7	6	4	9	11.2 ± 6.0	5.1 ± 2.4
	low	46.5 ± 11.8	51.7 ± 18.7	4	7	9	2	8	3	11.1 ± 5.4	6.2 ± 3.9
	p	0.0071	0.051	0.44		0.210		0.10		0.97	0.43
*PTEN* gain	high	55.6 ± 10.1	40.6 ± 16.7	2	3	2	3	2	3	15.3 ± 4.8	7.0 ± 2.9
	low	49.3 ± 14.1	46.8 ± 17.7	9	11	15	5	13	7	10.3 ± 5.7	5.8 ± 4.1
	p	0.28	0.49	1.00		0.28		0.36		0.83	0.50
pCR	yes	56 ± 15.0	39.5 ± 15.5	8	7	8	7	5	10	13.3 ± 5.7	6.8 ± 3.8
	no	48.9 ± 13.6	46.4 ± 20.0	4	10	12	2	11	3	9.9 ± 5.6	5.2 ± 3.7
	p	0.19	0.31	0.26		0.11		0.025		0.11	0.38

^a^ *p* probability calculated using Fischer’s Exact test on contingency tables (stage, ER IHC, PR IHC) or using t-test for comparison of age, tumor size, *ERBB2*/cell, and *ERBB2*/CEN17, as executed using JMP Statistical Software. All calculations use parameter cutoffs specified in Table 2

## Discussion

In an effort to better identify patients more likely to achieve pCR in patients with ERBB2-positive breast cancer, we have evaluated a series of additional gene and centromere probes as well as Ki-67 expression. In our cohort pCR was achieved in 52 % of patients. As part of the analysis of ISH results we have evaluated different parameters for describing abnormal gene and chromosome copy numbers. This is because there is no single parameter that best describes copy number for all genes and chromosomes for all tumors. For example, gene amplification is often defined as the presence of two or more copies of a gene per copy of the chromosome on which the gene normally resides. This definition of gene amplification was found to have strong clinical relevance with respect to prognosis [45] in breast cancers but has been applied widely to other genes and other cancers with little or no justification. In the present study we have also evaluated the ratio of various gene copy numbers to their respective chromosome copy numbers (as represented by the centromere copy number) and evaluated a wide range of cutoff values. As another measure of relative gene copy number, the percentage of cells with more gene than chromosome copies (gene/centromere gain), or less gene than chromosome copies (gene/centromere loss), or either more or less gene copies relative to their respective chromosomes (gene/centromere gain or loss) were evaluated. Additionally, we have looked at the percentages of cells with greater than two gene or chromosome copies (gene or chromosome gain), or less than two copies (gene or chromosome loss), or the sum of these abnormal copy numbers (gene or chromosome gain or loss). This series of parameters is similar to those used in previous studies that compared different copy number parameters for associations with patient diagnoses and/or outcomes in melanoma [46], esophageal cancer [47], lung cancer [48], and cervical cancer [49]. This is the first application of these parameters in cases with neoadjuvant treatment of ERBB2-positive breast cancer.

The importance of evaluating different parameters can be seen in Table 2 and Fig. 2 for the series of different parameters describing *MET* copy number relative to chromosome 7 copy number. In this series of parameters both gain of *MET* relative to chromosome 7 (AUC = 0.613, sensitivity = 0.58 and specificity = 0.67 at the optimal cutoff of 32 % cells with relative gain) and loss of *MET* relative to chromosome 7 (AUC = 0.651, sensitivity = 0.50 and specificity = 0.83 at the optimal cutoff of 45 % cells with relative loss) are associated weakly with pCR (*p* = 0.41 and 0.19, respectively at the optimal cutoffs). However, the sum of cells with relative gain and loss (*MET*/CEN7 gain or loss) is highly associated with pCR (AUC = 0.791, sensitivity = 0.92 and specificity = 0.67 at the optimal cutoff of 50 % cells with abnormal relative copy number with *p* = 0.0032). Furthermore, the average ratio of *MET*/CEN7 has no association with pCR, either high ratios or low ratios (*p* = 1.0 for either relationship at the optimal cutoffs), which is understandable since both *MET*/CEN7 gain (equating to higher ratio) and *MET*/CEN7 loss (equating to lower ratio) are associated with pCR and would tend to cancel each other in a ratio calculation. Therefore, proper parameter selection is very important since evaluation of these neoadjuvant specimens using a *MET*-to-CEN7 ratio, the most common measure of relative copy number, would have erroneously indicated a lack of prognostic value while use of the relative gene-to-chromosome imbalance parameter *MET*/CEN7 gain or loss provides a high statistical association. Similar to *MET* and chromosome 7, ISH data for the other genes and corresponding chromosomes were evaluated in terms of the various parameters defined in the Methods section of this paper. Of the other 3 gene probes only *PTEN* reached statistical significance and this was using *PTEN* gain in which patient tumors having higher numbers of cells with more than 2 copies of *PTEN* were associated with pCR (AUC = 0.674, sensitivity = 0.38 and specificity = 1.00 at the optimal cutoff of 58 % cells with *PTEN* gain, *p* = 0.039). Examining the actual ISH signals per cell and the average *PTEN*/CEN10 ratios, gain of *PTEN* was likely a result of chromosome 10 polysomy, with CEN10/cell ranging between 1.5 and 4.2 and *PTEN*/CEN10 not exceeding a ratio of 1.4 for any one specimen. Several specimens did exhibit *PTEN* deletion (*PTEN*/CEN10 < 0.7) but these appeared in both the pCR and non-pCR groups and no association was found for deletion using the parameters of *PTEN* loss, *PTEN*/CEN10 ratio, or *PTEN*/CEN10 loss.

Ki-67 expression, as measured by IHC, was also highly associated with pCR (AUC = 0.726, sensitivity = 1.00 and specificity = 0.42 at the optimal cutoff of 8 % cells expressing Ki-67, *p* = 0.0098). The only Ki-67 parameter evaluated was the percentage of cells expressing Ki-67, since this is a standard parameter used by pathologists in the evaluation of breast tissues. Since both *MET*/CEN7 gain or loss and Ki-67 parameters were highly associated with pCR, the two were analyzed in combination by holding *MET*/CEN7 gain or loss at its optimal cutoff of 50 % and varying the Ki-67 cutoff over a wide range (Fig. 2 and Table 2). This improved the AUC, and the optimal Ki-67 cutoff (8 % cells) provided sensitivity and specificity of 92 % and 83 %, respectively, and reduced the *p*-value below either single parameter (*p* = 0.0006).

The association between pCR and MET/CEN7 gain or loss may be an important finding since common clinical and pathologic characteristics were not found to have statistically significant associations with pCR (Table 3) in the neoadjuvant setting tested here. Of interest, MET/CEN7 gain or loss had little association with ERBB2 gene status but was associated with patient age and trended with tumor size and PR expression.

Our observations do not provide a mechanistic understanding of the role of MET copy number alterations in predicting pCR in HER2-positive breast cancer. And, these findings need to be validated in a subsequent, larger study of MET copy number alterations in a similar patient population. In an updated expansion cohort including patients treated with pertuzumab, additional studies with immunohistochemistry for MET and other markers to interrogate related signaling pathways (e.g., PI3K/AKT pathway) could be informative. One hypothesis is that any alteration of the MET signaling pathway may have some relationship to developing a pCR in the two treatment groups (ACTH and TCH) analyzed in this study. Increased or decreased MET expression potentially resulting from MET gene copy number change might create a fragile condition sensitive to perturbation by therapeutics.

## Conclusions

Our results show that a predictive score based on *MET* gene copy number relative to chromosome 7 and Ki-67 expression is strongly associated with pCR in patients with ERBB2-positive tumors treated with neoadjuvant chemotherapy and ERBB2-targeted therapy. Although the use of pCR as a surrogate endpoint for event free (EFS) and overall survival (OS) remains controversial, the ability to predict which patients are most likely to achieve a pCR remains important for individual patient treatment decisions and future clinical trial design. Patients with a sufficiently low likelihood of a pCR who are not candidates for breast conservation at presentation may choose surgery followed by adjuvant chemotherapy and ERBB2-targeted therapy. These data from retrospective studies require validation in a larger, prospective study.

## Additional file

Additional file 1: Supplementary Data - In situ hybridization parameters calculated for individual patients as used for ROC and contingency analyses (Tables 2 and 3 and Figs. 2 and 3 in main article). (XLS 57 kb)

## Abbreviations

ASCO: American society of clinical oncology
AUC: Area under curve
CAP: College of American pathologists
CEN: Centromere
CISH: Chromogen in situ hybridization
FDA: Food and drug administration
FFPE: Formalin fixed paraffin embedded
FISH: Fluorescence in situ hybridization
IHC: Immunohistochemistry
ISH: In situ hybridization
NSABP: National surgical adjuvant breast and bowel project
P: Probability
pCR: Pathologic complete response
ROC: Receiver operating characteristic
SISH: Silver in situ hybridization
